# Mini review: possible role of the multi-theory model of health behavior change in designing substance use prevention and treatment interventions

**DOI:** 10.3389/fpubh.2024.1298614

**Published:** 2024-03-01

**Authors:** Manoj Sharma, Asma Awan, Sidath Kapukotuwa

**Affiliations:** ^1^Department of Social and Behavioral Health, School of Public Health, University of Nevada, Las Vegas, Las Vegas, NV, United States; ^2^Department of Internal Medicine, Kirk Kerkorian School of Medicine at UNLV, Las Vegas, NV, United States

**Keywords:** multi-theory model, innovative, initiation, sustenance, fourth-generation, behavior

## Abstract

Many behavior change theories have evolved over time. Originally, the first conceptions prioritized interventions based on information, such as raising awareness and transferring knowledge. Second-generation theories prioritize the development of skills and the promotion of awareness. The emergence of evidence-based techniques in the 1990s gave rise to third-generation theories such as the Theory of Planned Behavior and Social Cognitive Theory. Presently, fourth-generation trends amalgamate various components from multiple theories to implement accurate treatments, employing technology and emphasizing targeted behavior change. This paper aims to do a concise evaluation of the multi-theory model (MTM) of health behavior change interventions in the context of planning substance use prevention and treatment. The current area of intervention programs aimed at preventing and treating substance use may benefit from MTM, an innovative fourth-generation behavior change model. Tobacco, alcohol, and other drugs have all been the subjects of experimental, cross-sectional, and qualitative research. We have presented that additional research is required to compare MTM with knowledge-based therapies or interventions grounded in other theories. A gold standard would be the randomized controlled trials and behavioral change interventions particularly useful for this purpose. In addition, research evaluating the interventions’ efficacy must be carefully planned and executed.

## 1 Introduction

Behavior change theories have developed over time. Initially, first-generation theories focused on knowledge-based interventions, including awareness building and knowledge transfer (1). Second-generation theories emphasize skill-building and consciousness-raising (2). Third-generation theories, like the Theory of Planned Behavior and Social Cognitive Theory, emerged in the 1990s as evidence-based approaches. Currently, fourth-generation trends integrate multiple constructs from multiple theories for precise interventions, utilizing technology and focusing on specific behavior change (3). The purpose of this article is to conduct a mini-review of the multi-theory model (MTM) of health behavior change interventions in designing substance use prevention and treatment. The mini review included search from databases: Medline (PubMed), CINAHL, and Scopus.

## 2 Multi-theory model of health behavior change

The multi-theory model (MTM) of health behavior change is an innovative fourth-generation model integrating insights from various behavioral theorists (3). It has two main components: initiation (one-time behavior change) and sustenance (maintaining behavior over time). MTM comprises robust constructs drawn from various theories from all four domains of cognition, conation, volition, and environment making it quite comprehensive. Initiation or starting a behavior change consists of three constructs. Firstly, *participatory dialogue* encourages two-way communication to discuss health behavior change’s pros and cons, based on Freire’s adult education model (4) and value expectancy theories (3). Secondly, *behavioral confidence* expands on Bandura’s self-efficacy (5) and Ajzen’s perceived behavioral control (6), emphasizing belief in one’s ability to enact the desired behavior while the sources of this surety arise from forces beyond the self. Thirdly, *changes in the physical environment* are influenced by Bandura’s concept of the environment (5), Prochaska’s environmental reevaluation (7), and Fishbein’s environmental factors (8). The sustenance component also consists of three constructs. Firstly, *emotional transformation*, based on Goleman’s (9) emotional intelligence theory, involves effectively managing emotions to sustain the behavior. Secondly, *practice for change*, influenced by Freire’s adult education model’s praxis (4), underscores the significance of consistent practice and behavior implementation. Lastly, *changes in the social environment* include Bandura’s (1986) concept of the environment, Prochaska’s (7) helping relationships, and social support (10), among others.

## 3 Applications of MTM

The MTM is versatile, finding applications across qualitative, quantitative, and experimental studies. For instance, Agyei-Baffour et al. (11) employed the MTM in a qualitative study to explore Ghanaian healthcare providers’ perceptions of human papillomavirus vaccination. Our research group also investigated college students’ yoga behavior recently (12). Over 50 cross-sectional studies, utilizing the MTM, have addressed a diverse array of health behaviors, encompassing practices like tooth-brushing, physical activity during pregnancy, dietary habits, and vaccine hesitancy (13–17). Similarly, the MTM has been employed in experimental studies aiming to enhance healthy behaviors, such as reducing sugar intake, improving the quality of life for postmenopausal women, and promoting increased consumption of fruits and vegetables (18–20), among other endeavors.

## 4 Applications of MTM in substance use prevention and treatment

As mentioned earlier, MTM addresses health behavior change into two stages derived from its phases *viz.*, initiation of the behavior change and sustenance or continuation of the health behavior change (Table 1). Implications of this theory have been explained as processes from moderation to responsible drinking, and/or abstinence in binge drinking in college students (23). This theory has also achieved its consistent parsimony in addressing substance use disorders and water pipe smoking since its core concepts have been tried and tested with positive results in other contexts (27, 28). Empirical support for MTM concepts can be utilized in college students who binge drink, to instill the aim of moderate drinking or abstinence. Interventions to promote safe drinking among this demographic group might benefit from this prediction model (24). Results from a qualitative suggested that MTM might be used to explain the water pipe smoking (WPS) reduction in the majority of high school students who agreed that lowering their WPS would have positive effects on their health, wallets, and reputations, as seen by their responses on the MTM-based survey (31). Prevention and simultaneous treatment (Table 2) can also be considered through the lens of the MTM components to the prediction of smoking initiation and sustenance behaviors among residents of a rural Kentucky county (25, 26).

**Table 1:** Descriptive and cross-sectional studies utilizing Multi-Theory Model (MTM) of health behavior change directed toward substance use treatment and prevention.

Author (Year)	Location of study	Target population	Type of behavior studied
(21)	Large University in the Southern United States	114 participants	Smoking cessation behavior; instrument development
(22)	A Southern University in United States	217 college students	Changing binge drinking to responsible drinking; instrument validation
(23)	Universities in the Southern United States	College students or persons aged 18–24 years	Responsible drinking or abstinence behavior; presented face and content validity of instrument
(24)	Large University in the Southern United States	289 undergraduate and graduate students	Binge drinking, responsible drinking, and/or abstinence behavior
(25)	Middlesboro, Kentucky	Then-current smokers participated voluntarily	Smoking cessation behavior
(26)	Rural, Appalachian Kentucky, United States	148 participants who smoked cigarettes, 18 years and older	Cigarette smoking cessation behavior
(27)	Hamadan city, Iran	200 high school students from grades 10–12	Waterpipe smoking reduction behavior
(28)	Substance use treatment facility	93 participants from the substance use treatment facility	Substance use change behavior
(29)	Kathmandu Metropolitan, Nepal	132 then-current smokers aged 16 years and over	Smoking cessation behavior
(30)	National representative sample in United States	619 young adults aged 18–24 years	

**Table 2:** Qualitative studies utilizing the Multi-Theory Model (MTM) of health behavior change directed toward substance use treatment and prevention.

Author (Year)	Location of study	Type of behavior studied	Study characteristics
(31)	Hamadan city, Iran	Water pipe smoking	34 interviews with first- and second-grade high school male students. Qualitative study utilized directed content analysis.
(32)	Hamadan City, Iran	Drug addiction	17 semi-structured and individualized interviews from male participants referred to drug addiction treatment centers. Qualitative study utilized theory-based directed content analysis.
(33)	Karachi, Pakistan	Smoking cessation and associated factors	13 in-depth, face-to-face, and semi-structured interviews from adult smokers with cardio-vascular or respiratory diseases. Qualitative descriptive exploratory study utilized a manual thematic analysis

**Figure 1:**
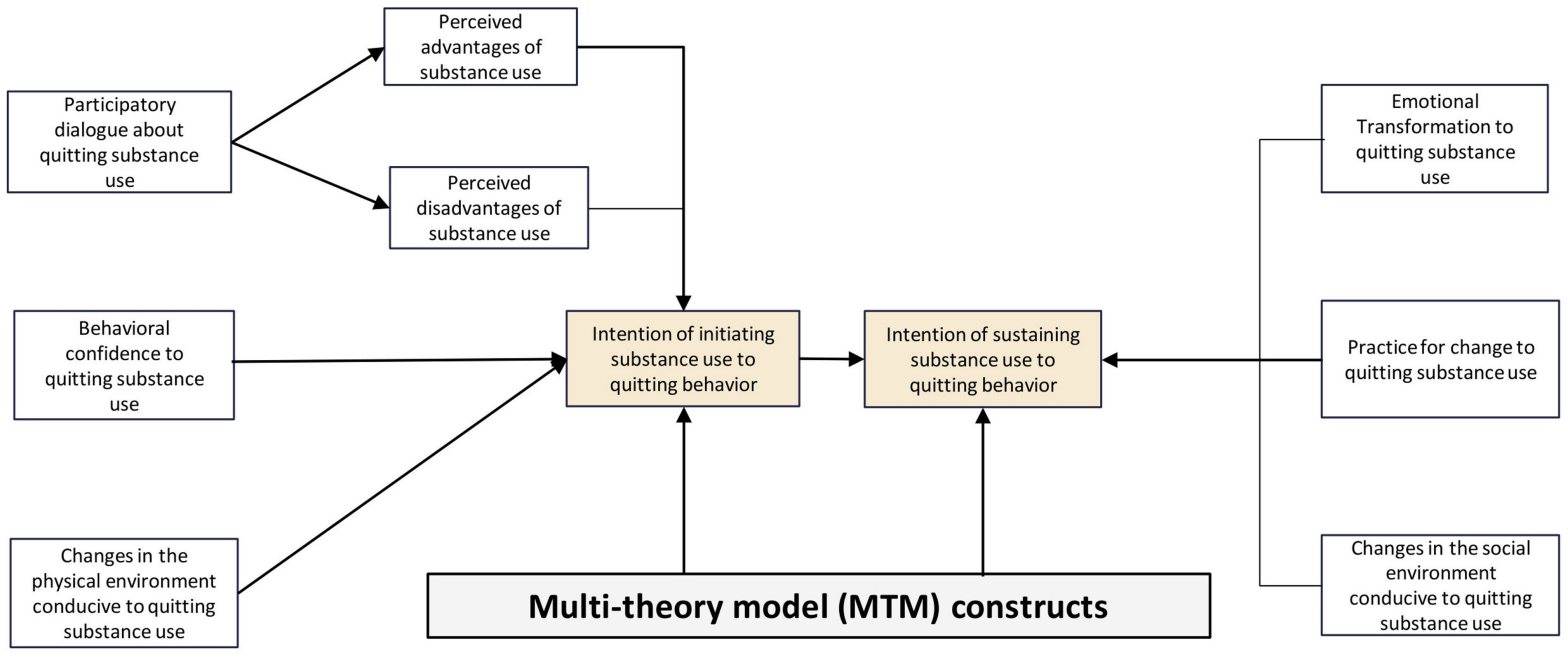
Application of multi-theory model of health behavior change for substance use quitting behavior.

MTM also provides a robust and evidence-based framework that proves valuable in the design of interventions aiming to improve responsible drinking or abstinence behaviors in binge drinking for college students (22, 24). It is important to develop and test educational interventions (Table 3) to see how well they work in different settings where young adults can be reached (31). The MTM seems suitable and ready to be used in the development of vaping cessation programs for young people (30), designing public smoking cessation programs, and tailoring smoking cessation strategies within the cultural contexts of the smoking population (21, 33), and decreasing the prevalence of smoking in the communities, colleges, and hospitals (29). The constructs of MTM have been verified in informing its usefulness in the design of interventions to prevent drug use and relapse affected by unpleasant emotions, family disputes, and access to drugs (32) and are considered significant factors in creating interventions within the personal and social dimensions to reduce water pipe smoking (WPS) among students influencing their own confidence, peers’ encouragement, and awareness of benefits of reducing WPS (31). The constructs also scored high in tobacco cessation and counseling programs measured from baseline and after 12 weeks of intervention (34) and implied success in alcohol and drug education to alter negative behaviors (35, 36). Figure 1 depicts the applicability of MTM in quitting substance use behavior.

**Table 3:** Experimental Studies Utilizing Multi-Theory Model (MTM) of Health Behavior Change directed toward substance use treatment and prevention interventions.

Author (Year)	Location of study	Participants	Type of study/design	Type of behavior studied	Outcomes
(31)	Hamadan city, Western Iran	94 male adolescent students; from grades 10 and 11	Randomized controlled trial (RCT), with a pretest-posttest design	Reducing water pipe smoking	Intervention was effective in reducing water pipe smoking
(34)	Bangalore city, India	64 tobacco product users visited or referred to Department of Public Health Dentistry	Non-randomized, uncontrolled trial design	Using MTM-focused intervention in tobacco cessation counseling (TCC)	Tobacco cessation counseling (TCC) was effective on follow-up scores

## 5 Limitations of MTM

MTM is an evolving model that will lead the path to fifth-generation models. One of the limitations of MTM is the variability in predictive power. Most of the cross-sectional studies have utilized multiple regression and the proportion of variance explained by this model for initiation and sustenance has varied from approximately 15 to 60% in various studies. Often not all the constructs are significant for different behaviors (37). Future research needs to reify the constructs better and improve upon the measurement. Also, besides multiple regression modeling, the evolving machine learning methods need to be applied to enhance predictive modeling. Another aspect suggested by some social psychologists is that of including the construct of *changes in the social environment* as a predictor in the initiation model. This idea is worthy of testing by future researchers. In experimental studies, the utilization of technology with MTM has been rather stunted. Future researchers must develop apps and other technological advancements to enhance the precision delivery of behavior change interventions based on MTM.

## 6 Conclusion

MTM is an advancing fourth-generation behavior change model that has potential applications for substance use prevention and treatment programs. Some qualitative, cross-sectional, and experimental studies have been done with alcohol, tobacco, and other drugs. However, more studies, especially those utilizing randomized controlled trials, and comparing MTM with either knowledge-based interventions or interventions based on other theories need to be conducted. Further, the interventions need to be scaled up and effectiveness studies need to be designed and tested.

## Author contributions

MS: Writing – original draft, Writing – review & editing, Conceptualization, Methodology, Supervision. AA: Writing – review & editing, Writing – original draft, Data curation, Investigation, Resources. SK: Writing – original draft, Writing – original draft, Data curation, Investigation, Resources.
